# From Airways to Alveoli: A Review of Smoking-Related Respiratory Disorders

**DOI:** 10.7759/cureus.104440

**Published:** 2026-02-28

**Authors:** Aya H Rudwan, Elaf Al Adhreai, Heba E Ali, Samaa Albaik, Yara Allo, Reem Osman, Yagen Bakhiet, Ahmed Rudwan

**Affiliations:** 1 Medicine, Istanbul Okan University, Istanbul, TUR; 2 Medicine and Surgery, Istanbul Okan University, Istanbul, TUR; 3 Medicine and Surgery, Ahfad University for Women, Khartoum, SDN; 4 Clinical Research, Hamad Medical Corporation, Doha, QAT; 5 Research, Hamad Medical Corporation, Doha, QAT

**Keywords:** lung cancer, nicotine use, respiratory disorder, smoking, smoking cessation

## Abstract

Despite significant declines in smoking rates over recent decades, tobacco use remains a leading preventable cause of respiratory illness and death worldwide. While global smoking rates have declined, progress has slowed in many regions, and the smoking-attributable disease burden continues to rise in low- and middle-income countries due to population growth and ageing. Smoking is a major risk factor for a wide range of respiratory diseases, including chronic obstructive pulmonary disease, lung cancer, and emerging post-infectious conditions, and it contributes significantly to disability-adjusted life years globally. This narrative review synthesizes the available literature to critically evaluate the impact of smoking on respiratory health, integrating evidence on conventional cigarette smoking and electronic cigarette use. It examines current trends, highlights unmet clinical and public health needs, and identifies key knowledge gaps to inform future research and tobacco control strategies. The review examines the respiratory effects of tobacco and e-cigarette constituents, including their inflammatory, oxidative, and immunomodulatory mechanisms, and assesses the physiological and clinical benefits of smoking cessation. Current pharmacological and behavioral cessation strategies are reviewed, alongside emerging approaches and public health interventions. Particular attention is given to disparities in respiratory health priorities, healthcare infrastructure, and access to evidence-based therapies across different socioeconomic settings. Finally, key knowledge gaps are identified, including limitations in long-term outcome data, underrepresentation of vulnerable populations, and insufficient evidence on newer nicotine products, and future research directions are proposed to strengthen tobacco control policies and improve respiratory disease prevention globally.

## Introduction and background

In 2019, approximately 1.14 billion people worldwide (95% uncertainty interval: 1.13-1.16 billion) were current smokers, consuming an estimated 7.41 trillion cigarette-equivalents of tobacco (7.11-7.74 trillion). Despite a notable decline in smoking prevalence since 1990-27.5% (26.5-28.5) among men and 37.7% (35.4-39.9) among women aged 15 years and older, population growth has caused the overall number of smokers to rise from 0.99 billion (0.98-1.00 billion) in 1990. In 2019, tobacco smoking was responsible for 7.69 million deaths (7.16-8.20 million) and 200 million disability-adjusted life-years (185-214 million), representing the leading cause of death among men (accounting for 20.2% (19.3-21.1) of male deaths). Of these deaths, 6.68 million (86.9%) occurred in current smokers [1].

Addressing the world’s respiratory health priorities in the twenty-first century is a complex and urgent challenge that demands coordinated action from individuals, communities, governments, healthcare systems, stakeholders, and international organizations [2]. Across many respiratory diseases, common risk factors include smoking, gender, and socioeconomic status. Although the extent to which these factors are modifiable remains debated, they nevertheless represent important targets for intervention. There are clear differences in respiratory health priorities across low-, middle-, and high-income countries (LMICs), many of which have been outlined in position papers on conditions such as chronic obstructive pulmonary disease (COPD) and long coronavirus disease (COVID). However, several overarching challenges cut across the respiratory diseases, including the need for higher-quality healthcare systems and more robust biomarker and risk-factor data from diverse regions. A major limitation in many LMICs is the lack of electronic medical records, which restricts the ability to determine temporal relationships between respiratory conditions. In addition, access to medications, particularly appropriate, evidence-based therapies, remains problematic in many parts of the world. Public awareness and patient education, including correct inhaler use, are also significant challenges, especially in rural areas of LMICs [2].

Given the evolving landscape of tobacco use, including the emergence of electronic nicotine delivery systems and persistent global disparities in smoking-related disease burden, a comprehensive understanding of smoking-related respiratory disorders remains essential. Despite extensive research, important gaps persist in long-term outcome data, representation of vulnerable populations, and evaluation of newer nicotine products.

This review aims to contextualize existing evidence within current clinical and public health challenges, emphasizing disease mechanisms, prevention strategies, and cessation approaches across diverse socioeconomic settings. By highlighting unresolved questions and unmet needs, this work seeks to inform future research directions and to support more effective respiratory disease prevention and tobacco control efforts.

Relevant literature was identified through a narrative search of electronic databases, including PubMed, Scopus, and Web of Science. Peer-reviewed articles published in English were prioritized, including epidemiological studies, systematic reviews, and key clinical research focusing on smoking, electronic cigarettes, and respiratory health.

## Review

Composition of tobacco and tobacco smoke

Tobacco and tobacco smoke contain a highly complex mixture of more than 9,500 chemical compounds, many of which are known to be toxic or carcinogenic. Regulatory authorities, such as the U.S. Food and Drug Administration (FDA) and the World Health Organization, have identified numerous harmful and potentially harmful constituents (HPHCs) within tobacco products. The FDA’s HPHC list, established in 2012, includes 93 compounds, 79 of which are classified as carcinogenic.

Similarly, the International Agency for Research on Cancer (IARC) has classified 83 carcinogens in tobacco and tobacco smoke as of 2022. Differences between FDA and IARC classifications reflect variations in evaluation criteria and the ongoing expansion of toxicological evidence.

Recent surveillance studies indicate that levels of key tobacco carcinogens, including polycyclic aromatic hydrocarbons (PAHs) and tobacco-specific nitrosamines (TSNAs), have not shown consistent declining trends in cigarette or oral tobacco products. Substantial variability has been reported in nicotine content, product pH, and TSNA concentrations across different products.

These findings highlight the continued public health importance of monitoring tobacco constituents and enforcing regulatory measures aimed at reducing exposure to carcinogens and other harmful substances associated with tobacco use [3].

Pathophysiological effects of smoking on the respiratory system

The respiratory epithelium serves as the primary protective barrier against inhaled environmental pollutants and pathogenic microorganisms. Exposure to cigarette smoke can directly injure the airway epithelial barrier, affecting ciliated cells, goblet cells, basal cells, and submucosal secretory glands. Harmful constituents of cigarette smoke disrupt coordinated ciliary beating, promote excessive mucus production, and slow mucociliary clearance, creating favorable conditions for pathogen colonization and proliferation [4].

These functional disturbances are associated with smoke-induced alterations in the metabolic activity of human airway basal stem and progenitor cells, thereby compromising the normal renewal of the mucociliary epithelium. In addition, cigarette smoke weakens epithelial integrity, primarily through the disruption of intercellular junctions. Conventional cigarette smoking reduces cough reflex sensitivity in humans, which limits the effective removal of inhaled pathogens. Notably, cough sensitivity may begin to recover within approximately two weeks following smoking cessation [4].

Compounds present in tobacco smoke can directly disrupt normal lung cell function and exhibit pro-inflammatory, cytotoxic, mutagenic, and carcinogenic effects. Exposure to inhaled oxidants causes direct pulmonary injury and initiates inflammatory processes that further aggravate tissue damage. Oxidative stress is a key mechanism in the development of several inflammatory lung diseases, including asthma, COPD, idiopathic pulmonary fibrosis, cystic fibrosis, and acute respiratory distress syndrome. In individuals who smoke, the oxidative load is increased due to reactive oxygen and nitrogen species originating from both tobacco smoke itself and from activated inflammatory cells within the lungs, such as macrophages, epithelial cells, neutrophils, and T lymphocytes. Moreover, interactions between cigarette smoke and the epithelial lining fluid promote additional generation of reactive oxygen species, as both contain metal ions, including iron, that facilitate free radical formation. A shift in the oxidant-antioxidant balance toward excessive oxidant activity leads to direct damage of lipids, proteins, nucleic acids, and structural components of the lung extracellular matrix, such as elastin and collagen. Oxidative stress is also associated with increased apoptosis, impaired skeletal muscle function, excessive mucus production, and reduced steroid receptor binding and translocation. Elevated reactive oxygen species contribute to the inactivation of antiproteases, including α1-antitrypsin, while simultaneously activating metalloproteases, resulting in a protease-antiprotease imbalance that promotes lung matrix degradation [5].

Additionally, cigarette smoking reduces pulmonary levels of glutathione, one of the lung’s primary antioxidants. Alterations in cellular redox homeostasis trigger inflammatory responses by enhancing the respiratory burst in phagocytic cells, modulating intracellular signaling pathways, inducing chromatin remodeling through histone acetylation and deacetylation, and activating redox-sensitive transcription factors such as nuclear factor-κB and activator protein-1. These transcription factors regulate the expression of pro-inflammatory cytokines, including interleukin-8 (IL-8), interleukin-6 (IL-6), and tumor necrosis factor-α, thereby linking cigarette smoke exposure to altered cytokine production. Additional mechanisms by which cigarette smoke influences cytokine gene expression involve smoke-induced epigenetic alterations, including changes in DNA methylation, microRNA expression, and histone modification [5].

Smoking and upper respiratory tract diseases

Chronic Rhinitis and Sinusitis

Christensen et al. conducted a systematic review examining the effects of cigarette smoke exposure on the sinonasal tract and its role in the development of chronic rhinitis and sinusitis. Cigarette smoke causes direct chemical injury to the sinonasal mucosal epithelium, compromising barrier integrity and impairing mucociliary clearance. These changes facilitate abnormal bacterial colonization and biofilm formation, which promote persistent mucosal inflammation through increased cytokine production and complement activation. Collectively, epithelial damage, immune dysregulation, ciliary dysfunction, and biofilm development contribute to a self-perpetuating cycle of chronic inflammation and infection, particularly in individuals exposed to secondhand smoke, including pediatric populations [6].

A more recent systematic review and meta-analysis by Tan et al. demonstrated that cigarette smoking is significantly associated with an increased prevalence and higher odds of chronic rhinosinusitis. Smoking was identified as an important predisposing factor; however, the authors reported that smoking status did not significantly affect postoperative outcomes following functional endoscopic sinus surgery (FESS) [7].

Pharyngitis and Laryngitis

Local inflammatory response, characterized by increased immune activation and irritation of the vocal cord mucosa, is provoked by inhaled toxicants in cigarette smoke. Clinical laryngitis occurs when the larynx exhibits irritation, coughing, and persistent throat clearing because of mucosal changes [8]. The mucosal changes, such as altered mucous production and impaired muco-ciliary clearance, manifest because of smoking, which ultimately causes chronic inflammation in the larynx [8]. Smokers typically exhibit reduced microbial diversity in the pharynx, compared to non-smokers. When compromised in this way, the pharyngeal environment is vulnerable and more susceptible to inflammatory responses [9]. Overall, smoking plays a direct role in promoting inflammatory changes that manifest as laryngitis and pharyngitis, by direct mucosal irritation in the larynx and disturbed microbiota in the pharynx [8].

Increased Susceptibility to Infections

Smoking is a major, dose-dependent risk factor for upper respiratory tract infections, with higher incidence and recurrence observed across both adult and pediatric populations, including children and infants exposed to active or passive smoking [4]. It impairs airway defenses, immune function, and mucociliary clearance. It also promotes microbial virulence, antibiotic resistance, inflammation, fibrosis, and immune suppression [10]. Consequently, smoking is a significant yet preventable cause of upper respiratory tract infections, underscoring the importance of smoking cessation and reducing secondhand smoke exposure in vulnerable populations [4,10].

Chronic Obstructive Pulmonary Disease (COPD)

**Figure 1:**
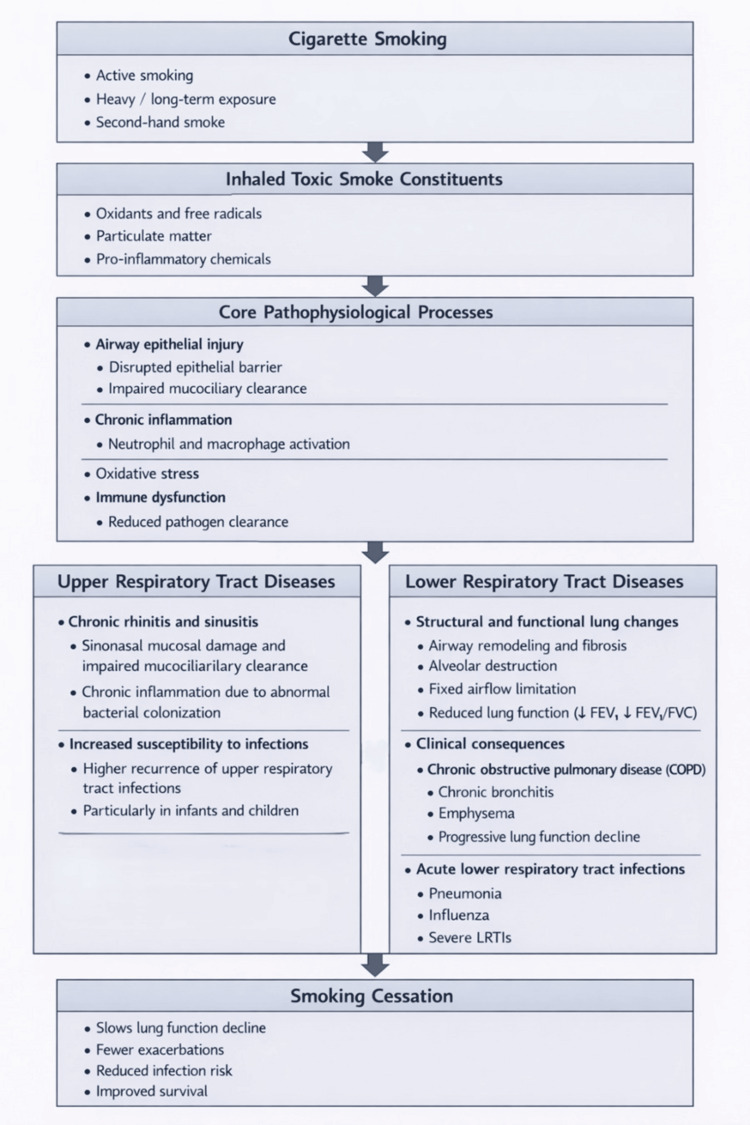
A conceptual overview of key pathophysiological mechanisms linking cigarette smoking to upper and lower respiratory tract diseases and the modifying effect of smoking cessation Source: [5,6,12,13] FVC: forced vital capacity; FEV1: forced expiratory volume in 1 s

Cigarette smoking is associated with an increased risk of COPD among young individuals, with current and heavy smokers exhibiting a higher likelihood of disease compared with non-smokers, and multivariate analysis in one nationwide cohort study found that female smokers had a relatively higher risk of COPD than male smokers after adjustment for confounders [11]. In a study that examined the impact of smoking behavior on the clinical course of COPD, patients were categorized according to mortality status and smoking cessation, forming a death group and a quitting‑smoking group. Death‑group patients exhibited a longer duration of smoking (40.3 ± 12.7 years vs. 35.7 ± 9.3 years), slightly older age of smoking onset (27.0 ± 8.8 years vs. 25.7 ± 7.4 years), and longer duration of smoking before COPD diagnosis (30.8 ± 13.0 years vs. 28.1 ± 10.0 years) compared with the survival group. They also had a lower prevalence of smoking cessation, later onset of COPD symptoms (57.5 ± 13.4 years vs. 53.3 ± 10.9 years), older age at quitting (62.8 ± 10.8 years vs. 55.6 ± 8.9 years), and lower forced expiratory volume in 1 second percentage (FEV₁%) predicted and FEV₁/forced vital capacity (FVC) ratios. The analysis demonstrated that age, age at smoking cessation, and FEV₁% predicted were independently associated with COPD‑related mortality and that the length of smoking exposure may contribute to mortality risk according to quantitative smoking indicators. Overall, these findings indicate that smoking cessation has the greatest impact on modifying the natural history of COPD, contributing to slower lung function decline, fewer symptoms and exacerbations, and improved survival (Figure 1) [12].

In a systematic review and meta-analysis evaluating the relationship between smoking and chronic respiratory diseases, it was found that smoking was strongly associated with an increased risk of COPD, chronic bronchitis, and emphysema [13].

Pneumonia and Acute Lower Respiratory Tract Infections

Evidence from a large prospective cohort study using data from the UK Biobank indicates that cigarette smoking is associated with an increased risk of severe acute infectious respiratory diseases. Among 341,352 participants without a prior history of major chronic disease, 12,384 cases of pneumonia, 7,054 cases of other acute lower respiratory tract infections, and 795 cases of influenza were recorded over a median follow-up of 12 years. Compared with never-smokers, current smokers had approximately two-fold higher hazards of severe infectious respiratory disease, including pneumonia (hazard ratio (HR) 2.40, 95% confidence interval (CI) 2.27-2.53), other acute lower respiratory tract infections (HR 1.99, 95% CI 1.84-2.14), and influenza (HR 1.82, 95% CI 1.47-2.24) [14]. In support of these findings, an evidence summary from the Centre for Evidence-Based Medicine (Oxford) highlights that cigarette smoking is a recognized risk factor for acute respiratory infections, increasing both susceptibility to infection and the likelihood of more severe clinical outcomes. The report also emphasizes that exposure to second-hand smoke contributes to acute respiratory infection risk and underscores the potential benefits of smoking cessation in reducing respiratory complications [15].

Smoking and asthma

Fu et al. demonstrated a clear association between asthma and smoking, especially in long-time smokers (over 40 years) and those who started smoking at a young age (under 18 years). Individuals who began smoking early and continued long-term had an approximately 65% higher risk of developing asthma compared with non-smokers. Early smoking was found to be an independent factor, meaning the increased risk remained even without accounting for the amount smoked. The relationship was found to be dose-responsive, with low smoke exposure correlating with a higher risk of asthma development, while high smoke exposure did not, due to its suppressive effect on allergic responses. This suggests that low-dose cigarette smoke is associated with allergic sensitization, while high-dose exposure may be associated with local allergic responses and instead contribute to the development of other diseases such as COPD [16].

Beiner et al. studied the association between smoking history and its impact on asthma severity [17]. The study was conducted on individuals with over 10 years of smoking history, who are often excluded from asthma studies. Their findings showed that smokers with more than 20 pack-years had significantly worse asthma control than non-smokers, with Asthma Control Test scores (ACT) 1.76 points lower than those of non-smokers. The ACT, Asthma Control Questionnaire-5 (ACQ-5), and mini Asthma Quality of Life Questionnaire (mini-AQLQ) are validated tools that assess symptom control, functional limitation, and quality of life in patients with asthma [17]. In smokers with asthma, lower scores on these questionnaires indicate poorer symptom control and reduced quality of life compared with non-smokers, as shown by reduced fractional exhaled nitric oxide (FeNO) levels. Despite these findings, inflammatory markers consistent with asthma were at similar levels between smokers and non-smokers with asthma, which suggests that treatment may not differ [18]. Chen et al. discussed the relationship between smoking and its impact on reduced ICS response. Their study showed that smokers with asthma required a higher inhaled corticosteroid (ICS) dose than non-smokers. It also demonstrated that asthmatic patients who quit smoking experienced reduced daytime and nighttime β2-agonist and ICS rescue use. Former smokers exhibited improved sensitivity to ICS and better overall asthma management. Long-term smoking was found to cause fixed airflow obstruction, contributing to decreased ICS responsiveness and necessitating higher ICS doses. Positive changes were observed following smoking cessation [19].

Smoking and lung cancer

In men living in Western nations, roughly 85% of lung cancer cases are attributed to cigarette smoking [20]. People who currently smoke or have smoked in the past face a substantially greater risk of lung cancer compared with those who never smoked, although this risk gradually declines the longer a person remains smoke-free [21]. Lung cancer rates have steadily declined in parallel with the long-term fall in smoking. Among Canadian men, smoking levels started to drop in the mid-1960s, and lung cancer incidence began to fall roughly two decades later. By 2010, the incidence had decreased to 38 cases per 100,000 men, compared with 60 per 100,000 in 1990. This downward trend mirrors the reduction in cigarette use, from an average of about 14 cigarettes per person per day in 1969 to approximately 9 per day by 1989. Overall, a 36% decrease in tobacco consumption corresponded to an estimated 37% decline in lung cancer diagnoses in men [22].

Each inhalation of cigarette smoke delivers a complex mixture of several thousand chemical constituents, more than 60 of which are recognized carcinogens. These cancer-causing agents span multiple chemical categories, including polycyclic aromatic hydrocarbons (PAHs), N-nitrosamines, aromatic amines, aldehydes, volatile organic compounds, and various metals. Beyond these well-characterized carcinogens, additional constituents, such as alkylated PAHs, oxidants, free radicals, and ethylating agents, have received comparatively limited investigation. Substantial evidence indicates that PAHs, N-nitrosamines, aromatic amines, and selected volatile organic compounds are among the primary contributors to smoking-related human cancers [23].

Experimental evidence from animal models indicates that exposure to e-cigarette aerosol can induce DNA damage and impair DNA repair mechanisms, leading to abnormal cellular proliferation and tumor development [24], suggesting carcinogenic effects driven by aerosol constituents beyond nicotine itself. Nicotine exposure directly activates nicotinic acetylcholine receptors, functioning as an agonist. This activation facilitates tumor proliferation, enhances cellular survival mechanisms, and promotes invasive behavior within malignant tissues [25]. Tobacco smoking is implicated in the etiology of all lung cancer subtypes, with the most significant associations observed in small-cell lung carcinoma and squamous cell carcinoma [23].

Passive smoking and health

Exposure to second-hand smoke (SHS), also known as passive or involuntary smoking, represents a significant public health risk for individuals who do not smoke. It is estimated that around 37% of the world’s population continues to be exposed to tobacco smoke, whether from the burning end of cigarettes or from exhaled smoke. Exposure is disproportionately higher among women and children than men, and significant disparities exist across racial and socioeconomic groups [26,27]. According to the 2019 Global Burden of Diseases, Injuries, and Risk Factors Study (GBD), second-hand smoke was responsible for an estimated 1.3 million (range: 1.0-1.6 million) deaths worldwide in 2019, with the highest impact observed in low- and middle-income countries [28].

Over recent decades, research on the health effects of SHS has advanced substantially, elucidating plausible biological mechanisms and critically evaluating the available evidence. Since the initial link to lung cancer reported in the 1986 Surgeon General’s report [29], SHS exposure has been causally associated with several adverse health outcomes in both adults and children, including cardiovascular disease, various respiratory conditions, middle ear infections, low birth weight, and sudden infant death syndrome [29,30]. Moreover, prior studies, including several meta-analyses, have provided indicative evidence supporting a potential link between exposure to second-hand smoke and breast cancer [31,32].

Electronic cigarettes and respiratory health

Composition of e-Cigarette Aerosols

An electronic cigarette (e-cigarette) typically consists of a cartridge or reservoir containing an e-liquid, most commonly a mixture of nicotine, propylene glycol (PG), and vegetable glycerin (VG), and a heating element designed to aerosolize this liquid. During inhalation, a sensor-activated microprocessor engages the heating element, producing a nicotine-containing aerosol that the user inhales. Flavoring agents, which can replicate traditional tobacco tastes as well as fruit or beverage flavors, are incorporated into the e-liquid. The PG-VG mixture serves as the primary solvent system to which nicotine and flavorings are added [33]. An experimental investigation of e-cigarette aerosol composition showed that individuals actively using e-cigarettes may inhale high concentrations of nicotine, averaging 146 ± 51 mg/m³ during extended vaping sessions. When e-liquid leaks through the cartridge filter, nicotine exposure increases substantially, reaching approximately 914 mg/m³. In contrast, nicotine levels detected in second-hand aerosol remain considerably lower, generally measuring below 1 mg/m³, with observed values ranging from about 0.43 to 1.74 mg/m³ [34].

Nicotine exposure from e-cigarettes is highly addictive and is of particular concern among adolescents and young adults, as it may disrupt brain development and increase the likelihood of sustained nicotine dependence [35]. In addition, experimental and clinical studies have demonstrated associations between e-cigarette use and airway inflammation, impaired lung function, and increased susceptibility to respiratory symptoms and infections [36]. Importantly, evidence suggests that e-cigarette use among individuals with no prior history of smoking may increase the risk of initiating combustible cigarette use, especially among youth, posing a significant public health concern [37]. Moreover, few longitudinal studies have been conducted to assess the potential adverse effects of e-cigarettes [38]. The wide variety of e-cigarette devices and the differing compositions of e-liquids add further complexity to evaluating their safety compared to traditional smoking [39]. Additionally, the variation in product standards complicates the ability to make general conclusions about e-cigarettes [40].

*Vaping-Associated Pulmonary Injury* *(VAPI)*

VAPI, also referred to as e-cigarette or vaping product use-associated lung injury (EVALI), is an acute or subacute respiratory condition that presents with a range of clinical and pathological features resembling those of multiple pulmonary disorders [41]. Based on CDC guidelines, EVALI is diagnosed clinically and requires a history of e-cigarette use within the 90 days prior to symptom onset, evidence of pulmonary infiltrates on a chest X-ray or CT scan, and the exclusion of alternative causes, such as infections [42].

While the exact cause of EVALI has not yet been determined, multiple potential factors are being explored. Among these, vitamin E acetate is the most widely implicated substance linked to e-cigarette or vaping product use-associated lung injury. This association is supported by a recent study in which vitamin E acetate was detected in the bronchoalveolar lavage (BAL) fluid of 48 out of 51 EVALI patients, whereas it was absent in samples from healthy control participants [43]. The underlying pathology of EVALI remains largely unclear, and a complete pathophysiological explanation for the lung damage observed in affected patients has not yet been determined. In a recent study, Butt et al. reported a broad range of histopathological features associated with EVALI, including acute fibrinous pneumonitis, diffuse alveolar damage, and organizing pneumonia, typically centered around the bronchioles and often accompanied by bronchiolitis [44].

The clinical presentation of EVALI encompasses a variety of symptoms, most frequently respiratory, including cough, chest pain, and shortness of breath. Patients may also experience gastrointestinal symptoms, such as abdominal pain, nausea, vomiting, and diarrhea, as well as systemic signs like fever, chills, and weight loss. Data from 98 cases reported in 2020 indicate that most affected individuals were male (79%) with a median age of 21 years [45]. The primary treatment for EVALI is supportive care. This often involves providing supplemental oxygen, either through a nasal cannula or high-flow systems, to keep oxygen levels between 88 and 92%. The decision to admit a patient to the hospital or manage them as an outpatient depends on symptom severity. Hospitalization is recommended for patients with respiratory distress, existing lung-related conditions, or oxygen saturation below 95% on room air. In severe cases of low oxygen levels, mechanical ventilation may be necessary [41].

Benefits of smoking cessation on health

Smoking cessation has been shown to have a positive impact on respiratory health, demonstrating measurable improvements in lung function and a reduction in respiratory symptoms. A new study published in the International Journal of Chronic Obstructive Pulmonary Disease shows that after just one month of quitting smoking, former smokers experienced measurable improvements in both lung function and metabolism [46]. Specifically, the average FEV₁ increased by about 200 mL, and small‑airway flow (FEF 25/75) also improved--signs that airway obstruction and inflammation begin to reverse quickly. Patients reported fewer respiratory symptoms and less breathlessness, and their performance on a six‑minute walking test improved, indicating better exercise capacity and overall respiratory health. On the metabolic side, total cholesterol dropped, and vitamin D levels rose (without vitamin supplementation), suggesting cardiovascular and general‑health benefits beyond the lungs [46]. In short-duration smoking, quitting even for a month can begin to reverse lung damage and improve metabolic health, offering concrete gains in function and well‑being rather than just long‑term promise [46].

Over time, sustained smoking cessation leads to a gradual reduction in cardiovascular mortality risk, approaching the level observed in individuals who have never smoked. Longitudinal data indicate that the probability of developing cardiovascular disease (CVD) continues to decline with increasing duration of abstinence from smoking. Within approximately five years of quitting, the risk of coronary artery disease and stroke is markedly lower compared to those who continue smoking. With 10-15 years of continuous abstinence, many former smokers exhibit cardiovascular mortality rates that approximate those seen in never‑smokers [47].

A systematic review and meta-analysis assessed 19 observational studies (4 case-control and 15 cohort studies) and explored how reducing daily cigarette consumption influences health risks compared to continuing heavy smoking. The pooled results indicate that substantial smoking reduction is associated with a lower risk of lung cancer. Specifically, reducing cigarettes per day (CPD) by more than half was linked to a relative risk (RR) of 0.72 (95% CI: 0.52-0.91), reducing from heavy to moderate smoking corresponded to an RR of 0.66 (95% CI: 0.46-0.85), and reducing from heavy to light smoking yielded the lowest RR of 0.60 (95% CI: 0.49-0.72) [48].

Smoking cessation strategies

Behavioral Interventions

Behavioral interventions aimed at smoking cessation are highly heterogeneous in terms of content, delivery format, and accessibility. Such interventions often include direct advice to stop smoking, practical guidance on quitting methods, or both, but may differ substantially in the theoretical models, behavior‑change techniques, intensity, and mode of delivery employed [49]. They may consist of a single brief advice session delivered by a health‑care professional [50] or provision of a printed leaflet [51], or they may involve more intensive programs, such as multiple counselling sessions [52]. In some cases, these more comprehensive interventions are augmented with additional components, for example, financial incentives or support from partners [53].

Pharmacological Smoking Cessation

A variety of pharmacological options are available for the management of tobacco dependence. These treatments are most effective when combined with evidence-based non-pharmacological interventions, such as behavioral counselling, which play a central role in supporting the cessation process. The primary medications used to aid smoking cessation include nicotine replacement therapy (NRT), varenicline, bupropion, cytisine [54], and nicotine antagonists like mecamylamine. Clinicians should base treatment selection on the best available scientific evidence while also considering the patient’s individual preferences. Careful evaluation is essential for individuals with conditions that may contraindicate certain therapies, particularly those with cardiovascular, renal, hepatic, or psychiatric comorbidities [55].

Evidence indicates that the combination of NRT and varenicline offers equivalent levels of effectiveness, and each outperforms single-form NRT and bupropion in promoting smoking cessation [56]. Recent network meta-analyses have shown that most pharmacological treatments, whether used alone or in combination, provide a clear advantage over placebo in supporting smoking cessation. Among single-agent therapies, varenicline demonstrated the strongest evidence of effectiveness. Additionally, the analyses indicated that certain combination regimens, including varenicline with bupropion and nicotine replacement therapy with mecamylamine, are among the interventions most likely to yield the highest cessation success rates [57].

Public health approaches to smoking cessation

Smoking cessation is a major public health priority because stopping smoking dramatically reduces the risk of disease and early death, regardless of age. Although many people who smoke express a desire to quit, most do not use proven treatments. This gap underscores the need for a broad, coordinated public health strategy that increases access to effective support and encourages more quitting attempts. A core element of the public health strategy is the integration of evidence-based treatments across the healthcare system. Behavioral counseling and FDA-approved cessation medications are highlighted as core tools that significantly improve the chances of quitting, especially when used together. The report also notes that telephone quit-lines and digital tools, such as interactive websites and text-based programs, can reach large segments of the population and provide support outside traditional clinical settings [55]. The public health approach also calls for stronger healthcare system structures that routinely address tobacco use. This includes insurance policies that remove financial barriers to cessation treatment, health systems that automatically screen for tobacco use, and performance measures that encourage clinicians to consistently offer cessation support. These system-level components aim to make quitting assistance a standard part of every healthcare encounter [55].

At the population level, population-level strategies help shift the environment toward one that promotes quitting. Increasing tobacco prices, implementing comprehensive smoke-free policies, sustaining large-scale media campaigns, and funding robust state tobacco control programs are identified as effective ways to motivate quit attempts and lower smoking rates across communities. Measures such as strong warning labels and readily accessible state quit lines further reinforce efforts to quit [55]. An effective public health approach to smoking cessation requires a comprehensive, multi-layered strategy. By combining clinical treatment, supportive health system policies, and population-level measures, communities can create conditions that encourage quitting, reduce tobacco-related disease, and improve health for the entire population.

## Conclusions

Smoking remains a major contributor to respiratory illness, accelerating the development of COPD, lung cancer, and other chronic lung conditions. Quitting smoking continues to be the most effective way to protect and improve respiratory health. Clinically, routine screening for tobacco use, brief counselling, and offering evidence-based cessation support are essential strategies for slowing disease progression and improving patient outcomes. Public health efforts, such as smoke-free policies, taxation, accessible cessation services, and clear communication about risks, are equally important for reducing smoking rates and preventing respiratory disease at the population level. Strengthening both clinical interventions and public-health measures will be crucial for reducing the burden of smoking-related respiratory illness and improving overall health outcomes.

However, critical gaps limit the effectiveness of current strategies: there is insufficient long-term evidence comparing traditional smoking with newer nicotine products, a limited understanding of nicotine’s direct effects, scarce research on alternatives like heated tobacco or snus, and an underrepresentation of vulnerable populations in studies. Addressing these gaps through rigorous, standardized, and inclusive research is crucial for improving respiratory health outcomes globally.
